# A phylogenetic and taxonomic study on *Steccherinum* (Polyporales, Basidiomycota): Focusing on three new *Steccherinum* species from southern China

**DOI:** 10.3389/fcimb.2022.1103579

**Published:** 2023-01-10

**Authors:** Jun-Hong Dong, Xun-Chi Zhang, Jia-Jia Chen, Zhong-Long Zhu, Chang-Lin Zhao

**Affiliations:** ^1^ Yunnan Key Laboratory of Plateau Wetland Conservation, Restoration and Ecological Services, Southwest Forestry University, Kunming, China; ^2^ College of Biodiversity Conservation, Southwest Forestry University, Kunming, China; ^3^ College of Landscape Architecture, Jiangsu Vocational College of Agriculture and Forestry, Zhenjiang, China; ^4^ College of Forestry, Beijing Forestry University, Beijing, China; ^5^ Yunnan Key Laboratory for Fungal Diversity and Green Development, Kunming Institute of Botany, Chinese Academy of Science, Kunming, China

**Keywords:** biodiversity, molecular systematics, Steccherinaceae, wood-inhabiting fungi, Yunnan Province

## Abstract

The wood-inhabiting fungi play an integral role in wood degradation and the cycle of matter in the ecological system. They are considered as the “key player” in wood decomposition, because of their ability to produce all kinds of enzymes that break down woody lignin, cellulose and hemicellulose. In the present study, three new wood-inhabiting fungal species, *Steccherinum fissurutum*, *S. punctatum* and *S. subtropicum* spp. nov., collected from southern China, are proposed based on a combination of morphological features and molecular evidence. *Steccherinum fissurutum* is characterized by the resupinate, subceraceous basidiomata with cracked hymenophore, a monomitic hyphal system with clamped generative hyphae and cylindrical basidiospores; *S. punctatum* is characterized by the annual, punctate basidiomata with leathery hymenophore, cylindrical, strongly encrusted cystidia and ellipsoid basidiospores (3.6–4.5 ×2.6–3.4 µm); *S. subtropicum* is characterized by its effuse-reflexed basidiomata, a odontioid hymenophore with pink to lilac hymenial surface and ellipsoid basidiospores measuring as (2.8–3.4 × 2.0–2.7 µm). Sequences of ITS and nLSU rRNA markers of the studied samples were generated, and phylogenetic analyses were performed with maximum likelihood, maximum parsimony, and Bayesian inference methods. The ITS+nLSU analysis of the family Steccherinaceae indicated that the three new species clustered into the genus *Steccherinum*. Based on further analysis of ITS+nLSU dataset, the phylogenetic analysis confirmed that *S. subtropicum* was sister to *S*. *enuispinum*; *S*. *fissurutum* formed a monophyletic lineage; *S. punctatum* grouped with a clade comprised *S. straminellum* and *S. ciliolatum*.

## Introduction

The phylum Basidiomycota constitute a major group of the kingdom Fungi and is second in species numbers to the phylum Ascomycota (Wijayawardene et al., 2017; Wijayawardene et al., 2018a; Wijayawardene et al., 2018b). Wood-inhabiting fungal is a large group of Basidiomycota with simpler basidiomata with the diverse morphological features, but the phylogenetic diversity of this group is less intensively studied (Larsson et al., 2004; Bernicchia and Gorjón, 2010).

The genus *Steccherinum* Gray (Steccherinaceae, Polyporales), typified by *S. ochraceum* (Pers. ex J.F. Gmel.) Gray, was established by Gray (1821). It is a cosmopolitan genus characterized by a combination of resupinate to effused-reflexed or pileate basidiome with a membranaceous consistencey, hymenophore odontioid to hydnoid, a dimitic hyphal structure with clamp connections or simple-septate generative hyphae, cystidia numerous, strongly encrusted in the obtuse apex, basidia subclavate and basidiospores hyaline, thin-walled, smooth, ellipsoid to subcylindrical, acyanophilous and negative in Melzer’s reagent (Fries, 1821; Gray, 1821; Bernicchia and Gorjón, 2010). So far, about 80 species have been accepted in this genus worldwide (Fries, 1821; Banker, 1906; Banker, 1912; Cunningham, 1958; Snell and Dick, 1958; Lindsey and Gilbertson, 1977; Ryvarden, 1978; Lindsey and Gilbertson, 1979; Burdsall and Nakasone, 1981; Melo, 1995; Legon and Roberts, 2002; Yuan and Dai, 2005a; Spirin et al., 2007; Hjortstam and Ryvarden, 2008; Bernicchia and Gorjón, 2010; Miettinen et al., 2012; Yuan and Wu, 2012; Miettinen and Ryvarden, 2016; Westphalen et al., 2018; Liu and Dai, 2021; Westphalen et al., 2021; Wu et al., 2021a; Wu et al., 2021b; Dong et al., 2022). In recent years, several new *Steccherinum* species were described in China, *S. fragile* Z.B. Liu & Y.C. Dai, *S. hirsutum* Y.X. Wu & C.L. Zhao, *S. puerense* Y.X. Wu, J.H. Dong & C.L. Zhao, *S. rubigimaculatum* Y.X. Wu, J.H. Dong & C.L. Zhao, *S. subcollabens* (F. Wu, P. Du & X.M. Tian) Z.B. Liu & Y.C. Dai, *S. tenuissimum* C.L. Zhao & Y.X. Wu and *S. xanthum* C.L. Zhao & Y.X. Wu, and *S. yunnanense* Y.X. Wu & C.L. Zhao (Liu and Dai, 2021; Wu et al., 2021a; Wu et al., 2021b; Dong et al., 2022).

Molecular phylogenies have provided increased knowledge concerning the evolution of *Steccherinum* (Miettinen et al., 2012; Binder et al., 2013; Justo et al., 2017; Westphalen et al., 2018; Westphalen et al., 2021). Utilizing sequences of the gene regions ITS, nLSU, mtSSU, *atp6*, *rpb2*, and *tef1*, Miettinen et al. (2012) revealed that the phylogeny of the poroid and hydnoid genera *Antrodiella* Ryvarden and I. Johans., *Junghuhnia* Corda and *Steccherinum* (Polyporales, Basidiomycota) grouped together and *Steccherinum* was shown to contain both hydnoid and poroid species. Using of whole genome sequence data in comparison to extensively sampled multigene datasets indicated that *Steccherinum* species belonged to the residual polyporoid clade and the generic type (*S. ochraceum*) was grouped with *Junghuhnia nitida* (Pers.) Ryvarden (Binder et al., 2013). Justo et al. (2017) clarified family-level classification of eighteen families within the order Polyporales (Basidiomycota), which showed that *Steccherinum* belonged to family Steccherinaceae Parmasto. Westphalen et al. (2018) worked on morphological and multigene analyses of *Junghuhnia* s.lat., in which a new species *Steccherinum neonitidum* Westphalen & Tomšovský and three new combinations, *S. meridionale* (Rajchenb.) Westphalen, Tomšovský & Rajchenberg, *S. polycystidiferum* (Rick) Westphalen, Tomšovský & Rajchenb. and *S. undigerum* (Berk. & M.A. Curtis) Westphalen & Tomšovský were reported. Westphalen et al. (2021) provided the morphological and phylogenetic analyses on hydnoid specimens of Steccherinaceae, in which four genera as *Cabalodontia* Piatek, *Etheirodon* Banker, *Metuloidea* G. Cunn., and *Steccherinum* were introduced and three new neotropical species was found.

Scientific names are important link to communicate biological information across many spheres of use, in which how to publish a new fungal species is recommended to provide DNA barcode sequences in a public repository for the holotype specimen with the barcode locus (ITS) as well as any additional taxa specific secondary barcode loci (Aime et al., 2021). In order to allow BLAST searches to work optimally, sequences of DNA barcodes should include the generally used region for that marker (Aime et al., 2021). Sometimes, this genus *Steccherinum* for the barcoding gene ITS is less than 97% of nucleotide difference between different species.

The aim of this study is to explore the diversity and phylogeny of *Steccherinum* in China. During our investigations on the diversity of wood-inhabiting fungi in southern China, three undescribed species were collected from Yunnan Province, and their morphology corresponds to the concept of *Steccherinum*. To confirm their placement in *Steccherinum*, morphological examination and phylogenetic analyses based on the internal transcribed spacer (ITS) and large subunit nuclear ribosomal RNA (nLSU) genens, were carried out.

## Materials and methods

### Morphological studies

The studied specimens are deposited at the herbarium of Southwest Forestry University (SWFC), Yunnan Province, P.R. China (Herbarium numbers: *Steccherinum fissurutum*: SWFCF00021634, SWFCF00021673, SWFCF00021675, SWFCF00021680, SWFCF00021703, SWFCF00021744, SWFCF00021754, SWFCF00020803, SWFCF00021808, SWFCF00021811, SWFCF00021826, SWFCF00021841; *S. punctatum*: SWFCF00009181, SWFCF00009184; *S. subtropicum*: SWFCF00011059, SWFCF00016901). Macromorphological descriptions are based on field notes. Petersen (1996) was followed for the colour terms. Micromorphological data were obtained from the dried specimens and observed under a light microscope Eclipse E 80i (Nikon, Tokyo) following Dai (2012). The following abbreviations were used for the micro characteristics description: KOH = 5% potassium hydroxide, CB = Cotton Blue, CB– = acyanophilous, IKI = Melzer’s reagent, IKI– = both non-amyloid and non-dextrinoid, L = mean spore length (arithmetic average of all spores), W = mean spore width (arithmetic average of all spores), Q = variation in the L/W ratios between the specimens studied, n (a/b) = number of spores (a) measured from given number (b) of specimens.

### Molecular procedures and phylogenetic analyses

CTAB rapid plant genome extraction kit-DN14 (Aidlab Biotechnologies Co., Ltd, Beijing) was used to obtain genomic DNA from dried specimens, according to the manufacturer’s instructions. ITS region was amplified with primer pairs ITS5 and ITS4 (White et al., 1990). Nuclear LSU region was amplified with primer pairs LR0R and LR7 (https://sites.duke.edu/vilgalyslab/rdna_primers_for_fungi/ ) **Table 1**.

**Table 1:** A list of genes, primers and primer sequences used in this study.

Fragment of amplification	Name of primer	Primer base sequence (5′-3′) ^b^	References
ITS	ITS5	GGA AGT AAA AGT CGT AAC AAG G	White et al., 1990
	ITS4	TCC TCC GCT TAT TGA TAT GC	
nLSU	LR0R	ACC CGC TGA ACT TAA GC	http://www.biology.duke.edu/fungi/mycolab/primers.htm
	LR7	TAC TAC CAC CAA GAT CT	

b degenerate base: R = A or G, Y = C or T, N = A or T or C or G, V = G or A.

The PCR procedure for ITS was as follows: initial denaturation at 95°C for 3 min, followed by 35 cycles at 94°C for 40 s, 58°C for 45 s and 72°C for 1 min, and a final extension of 72°C for 10 min. The PCR procedure for nLSU was as follows: initial denaturation at 94°C for 1 min, followed by 35 cycles at 94°C for 30 s, 48°C 1 min and 72°C for 1.5 min, and a final extension of 72°C for 10 min. The PCR products were purified and sequenced at Kunming Tsingke Biological Technology Limited Company, Kunming, Yunnan Province, P.R. China. All newly generated sequences were deposited at GenBank (**Table 2**).

**Table 2:** List of species, specimens and GenBank accession numbers of sequences used in this study. * is shown type material, holotype.

Species Name	Sample No.	GenBank Accession No.		References
		ITS	nLSU	
*Antella americana*	KHL 11949	JN710509	JN710509	Cao et al., 2021
*A. americana*	HHB-4100	KP135316	KP135196	Cao et al., 2021
*A. chinensis*	Dai 8874	JX110843	KC485541	Yuan, 2013
*A. chinensis*	Dai 9019	JX110844	KC485542	Yuan, 2013
*A. niemelaei*	Renvall 3218	AF126876	—	Cao et al., 2021
*A. niemelaei*	Haikonen 14727	AF126877	—	Cao et al., 2021
*Antrodiella onychoides*	Miettinen 2312	JN710517	JN710517	Miettinen et al., 2012
*A. pallescens*	Nordén 8.8.2008	JN710518	JN710518	Miettinen et al., 2012
*A. romellii*	Miettinen 7429	JN710520	JN710520	Miettinen et al., 2012
*A. semisupina*	Labrecque & Labbé 372	JN710521	JN710521	Miettinen et al., 2012
*A. stipitata*	FD-136	KP135314	KP135197	Westphalen et al., 2021
*A. stipitata*	Yuan 5640	KC485525	KC485544	Yuan, 2014
*Atraporiella neotropica*	Miettinen X1021	HQ659221	HQ659221	Cao et al., 2021
*A. yunnanensis*	CLZhao 604	MF962482	MF962485	Wu et al., 2017
*A. yunnanensis*	CLZhao 605	MF962483	MF962486	Wu et al., 2017
*Butyrea japonica*	MN 1065	JN710556	JN710556	Cao et al., 2021
*B. luteoalba*	FP-105786	KP135320	KP135226	Dong et al., 2022
*B. luteoalba*	KHL 13238b	JN710558	JN710558	Dong et al., 2022
*Climacocystis borealis*	KHL 13318	JN710527	JN710527	Cao et al., 2021
*Elaphroporia ailaoshanensis*	CLZhao 596	MG231572	MG748855	Wu et al., 2018
*E. ailaoshanensis*	CLZhao 597	MG231847	MG748856	Wu et al., 2018
*Etheirodon fimbriatum*	KHL 11905	JN710530	JN710530	Cao et al., 2021
*E. fimbriatum*	HR 98811	MT849300	—	Westphalen et al., 2021
*E. purpureum*	MCW 642/18	MT849301	MT849301	Westphalen et al., 2021
*Flaviporus brownii*	MCW 362/12	KY175008	KY175008	Westphalen et al., 2018
*F. brownie*	X 462	JN710538	JN710538	Cao et al., 2021
*F. liebmannii*	X 249	JN710539	JN710539	Cao et al., 2021
*F. liebmannii*	Yuan 1766	KC502914	—	Yuan, 2014
*F. subundatus*	MCW 367/12	KY175004	KY175004	Westphalen et al., 2018
*F. subundatus*	MCW 457/13	KY175005	KY175005	Westphalen et al., 2018
*F. tenuis*	MCW 442/13	KY175001	KY175001	Westphalen et al., 2018
*F. tenuis*	MCW 356/12	KY175002	KY175002	Westphalen et al., 2018
*Frantisekia fissiliformis*	CBS 435.72	MH860521	MH872232	Vu et al., 2019
*F. mentschulensis*	BRNM 710170	FJ496670	FJ496728	Dong et al., 2022
*F. mentschulensis*	AH 1377	JN710544	JN710544	Dong et al., 2022
*F. ussurii*	Wei 3081	KC485527	KC485545	Yuan, 2014
*F. ussurii*	Dai 8249	KC485526	—	Yuan, 2014
*Irpex lacteus*	DO 421/951208	JX109852	JX109852	Dong et al., 2022
*Junghuhnia crustacea*	X 262	JN710553	JN710553	Miettinen et al., 2012
*J. delicate*	MCW 564/17	MT849295	MT849295	Du et al., 2020
*J. delicate*	MCW 693/19	MT849297	MT849297	Du et al., 2020
*J. pseudocrustacea*	Yuan 6160	MF139551	—	Yuan et al., 2019
*J. pseudocrustacea*	Zhou 283	MF139552	—	Yuan et al., 2019
*Loweomyces fractipes*	X 1149	JN710570	JN710570	Cao et al., 2021
*L. fractipes*	MT 13/2012	KX378866	KX378866	Cao et al., 2021
*L. spissus*	MCW 488/14	KX378869	KX378869	Cao et al., 2021
*L. tomentosus*	MCW 366/12	KX378870	KX378870	Cao et al., 2021
*L. wynneae*	X 1215	JN710604	JN710604	Cao et al., 2021
*Metuloidea cinnamomea*	X 1228	KU926963	—	Cao et al., 2021
*M. fragrans*	LE 295277	KC858281	—	Cao et al., 2021
*M. murashkinskyi*	X 449	JN710588	JN710588	Cao et al., 2021
*M. reniformis*	MCW 542/17	MT849303	MT849303	Westphalen et al., 2021
*M. reniformis*	MCW 523/17	MT849302	MT849302	Westphalen et al., 2021
*M. rhinocephala*	X 460	JN710562	JN710562	Cao et al., 2021
*Mycorrhaphium hispidum*	MCW 363/12	MH475306	MH475306	Cao et al., 2021
*M. hispidum*	MCW 429/13	MH475307	MH475307	Cao et al., 2021
*M. subadustum*	Yuan 12976	MW491378	MW488040	Cao et al., 2021
*M. subadustum*	Dai 10173	KC485537	KC485554	Cao et al., 2021
*Nigroporus stipitatus*	KaiR 116	MT110231	MT110231	Piepenbring et al., 2020
*N. vinosus*	MQN 015	AB811861	AB811861	Hai Bang et al., 2014
*N. vinosus*	X 839	JN710575	JN710575	Cao et al., 2021
*Steccherinum autumnale*	Spirin 2957	JN710549	JN710549	Liu and Dai, 2021
*S. bourdotii*	HR99893	MT849311		Westphalen et al., 2021
*S. bourdotii*	Saarenoksa 10195	JN710584	JN710584	Miettinen et al., 2012
*S. ciliolatum*	Ryvarden 47033	JN710585	JN710585	Miettinen et al., 2012
*S. collabens*	KHL 11848	JN710552	JN710552	Liu and Dai, 2021
***S. fissurutum***	CLZhao 21803 *	**OP799385**	**OP799397**	**Present study**
***S. fissurutum***	**CLZhao 21841**	**OP799388**	**OP799400**	**Present study**
***S. fissurutum***	**CLZhao 21808**	**OP799386**	**OP799398**	**Present study**
***S. fissurutum***	**CLZhao 21675**	**OP799380**	**OP799392**	**Present study**
***S. fissurutum***	**CLZhao 21811**	**OP799389**	**OP799399**	**Present study**
***S. fissurutum***	**CLZhao 21680**	**OP799381**	**OP799393**	**Present study**
***S. fissurutum***	**CLZhao 21703**	**OP799382**	**OP799394**	**Present study**
***S. fissurutum***	**CLZhao 21744**	**OP799383**	**OP799395**	**Present study**
***S. fissurutum***	**CLZhao 21826**	**OP799387**	**—**	**Present study**
***S. fissurutum***	**CLZhao 21634**	**OP799378**	**—**	**Present study**
***S. fissurutum***	**CLZhao 21673**	**OP799379**	**—**	**Present study**
***S. fissurutum***	**CLZhao 21754**	**OP799384**	**OP799396**	**Present study**
*S. fragile*	Dai 19972	MW364629	MW364627	Liu and Dai, 2021
*S. fragile*	Dai 20479	MW364628	MW364626	Liu and Dai, 2021
*S. hirsutum*	CLZhao 4222	MW290040	MW290054	Dong et al., 2022
*S. hirsutum*	CLZhao 4523	MW290041	MW290055	Dong et al., 2022
*S. larssonii*	MCW 593/17	MT849306	MT849306	Westphalen et al., 2021
*S. larssonii*	MCW 594/17	MT849307	MT849307	Westphalen et al., 2021
*S. meridionalis*	MR 10466	KY174994	KY174994	Westphalen et al., 2018
*S. meridionalis*	MR 284	KY174992	KY174992	Westphalen et al., 2018
*S. neonitidum*	MCW 371/12	KY174990	KY174990	Westphalen et al., 2018
*S. neonitidum*	RP 79	KY174991	KY174991	Westphalen et al., 2018
*S. nitidum*	KHL 11903	JN710560	JN710560	Westphalen et al., 2018
*S. nitidum*	MT 33/12	KY174989	KY174989	Westphalen et al., 2018
*S. ochraceum*	KHL11902	JN710590	JN710590	Westphalen et al., 2021
*S. ochraceum*	2060	JN710589	JN710589	Liu and Dai, 2021
*S. polycystidiferum*	RP 140	KY174996	KY174996	Westphalen et al., 2018
*S. polycystidiferum*	MCW 419/12	KY174995	KY174995	Westphalen et al., 2018
*S. pseudozilingianum*	Kulju 1004	JN710561	JN710561	Liu and Dai, 2021
*S. puerense*	CLZhao 3122	MW682341	—	Wu et al., 2021a
*S. puerense*	CLZhao 3644	MW682342	MW682338	Wu et al., 2021a
***S. punctatum***	**CLZhao 9181**	**OP799375**	**OP799401**	**Present study**
***S. punctatum***	CLZhao 9184 *	**OP799376**	**OP799402**	**Present study**
*S. robustius*	G1195	JN710591	JN710591	Cao et al., 2021
*S. rubigimaculatum*	CLZhao 4069	MW682343	MW682339	Wu et al., 2021a
*S. rubigimaculatum*	CLZhao 10638	MW682344	MW682340	Wu et al., 2021a
*S. straminellum*	KHL 13849	JN710597	JN710597	Cao et al., 2021
*S. subcollabens*	Dai 19344	MN871758	MN877771	Liu and Dai, 2021
*S. subcollabens*	Dai 19345	MN871759	MN877772	Liu and Dai, 2021
***S. subtropicum***	**CLZhao 16901**	**OP799391**	**—**	**Present study**
***S. subtropicum***	CLZhao 11059 *	**OP799390**	**OP799377**	**Present study**
*S. tenue*	FP-102082	KY948817	—	Liu and Dai, 2021
*S. tenue*	KHL 12316	JN710598	JN710598	Liu and Dai, 2021
*S. tenuispinum*	Spirin 2116	JN710600	JN710600	Miettinen et al., 2012
*S. tenuispinum*	Miettinen 8065	JN710599	JN710599	Miettinen et al., 2012
*S. undigerum*	MCW 472/13	KY174987	KY174987	Westphalen et al., 2018
*S. undigerum*	MCW 426/13	KY174986	KY174986	Westphalen et al., 2018
*S. xanthum*	CLZhao 5030	MW204588	MW204577	Wu et al., 2021b
*S. xanthum*	CLZhao 5032	MW204589	MW204578	Wu et al., 2021b
*S. yunnanense*	CLZhao 1445	MW290042	MW290056	Dong et al., 2022
*S. yunnanense*	CLZhao 2822	MW290043	MW290057	Dong et al., 2022
*Trullella conifericola*	Cui 2851	MT269764	—	Cao et al., 2021
*T. conifericola*	Yuan 12655	MT269760	MT259326	Cao et al., 2021
*T. dentipora*	X 200	JN710512	JN710512	Cao et al., 2021
*T. duracina*	MCW 410/13	MH475309	MH475309	Cao et al., 2021
*T. duracina*	RP 96	MH475310	MH475310	Cao et al., 2021
*Xanthoporus syringae*	Jeppson 2264	JN710607	JN710607	Cao et al., 2021
*X. syringae*	AFTOL-ID 774	AY789078	AY684166	Cao et al., 2021

* is shown type material, holotype.

**Figure 1:**
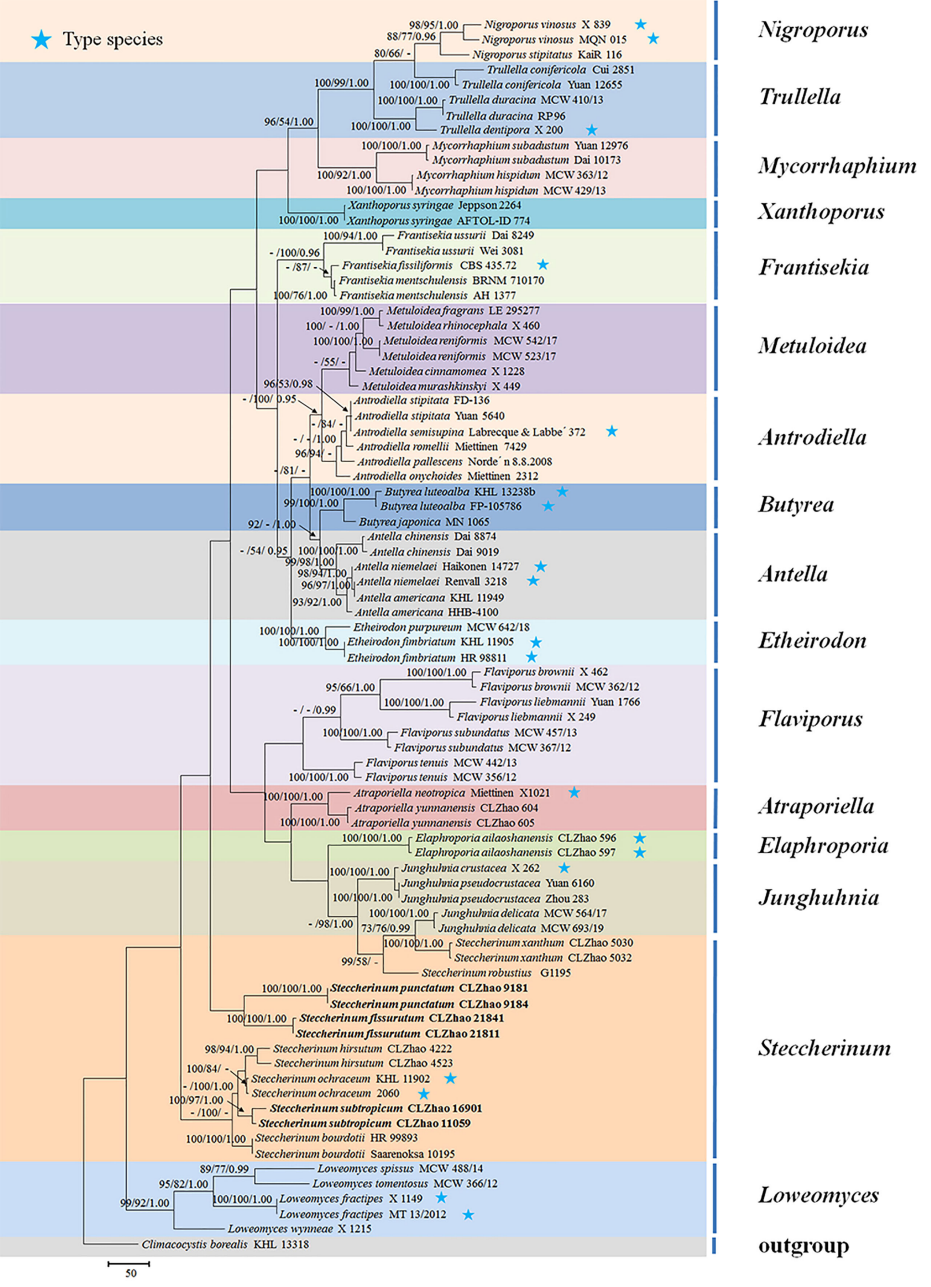
Maximum parsimony strict consensus tree illustrating the phylogeny of three new species and related species in the family Steccherinaceae based on ITS+nLSU sequences. Branches are labeled with maximum likelihood bootstrap values higher than 70%, parsimony bootstrap values higher than 50% and Bayesian posterior probabilities more than 0.95 respectively.

**Figure 2:**
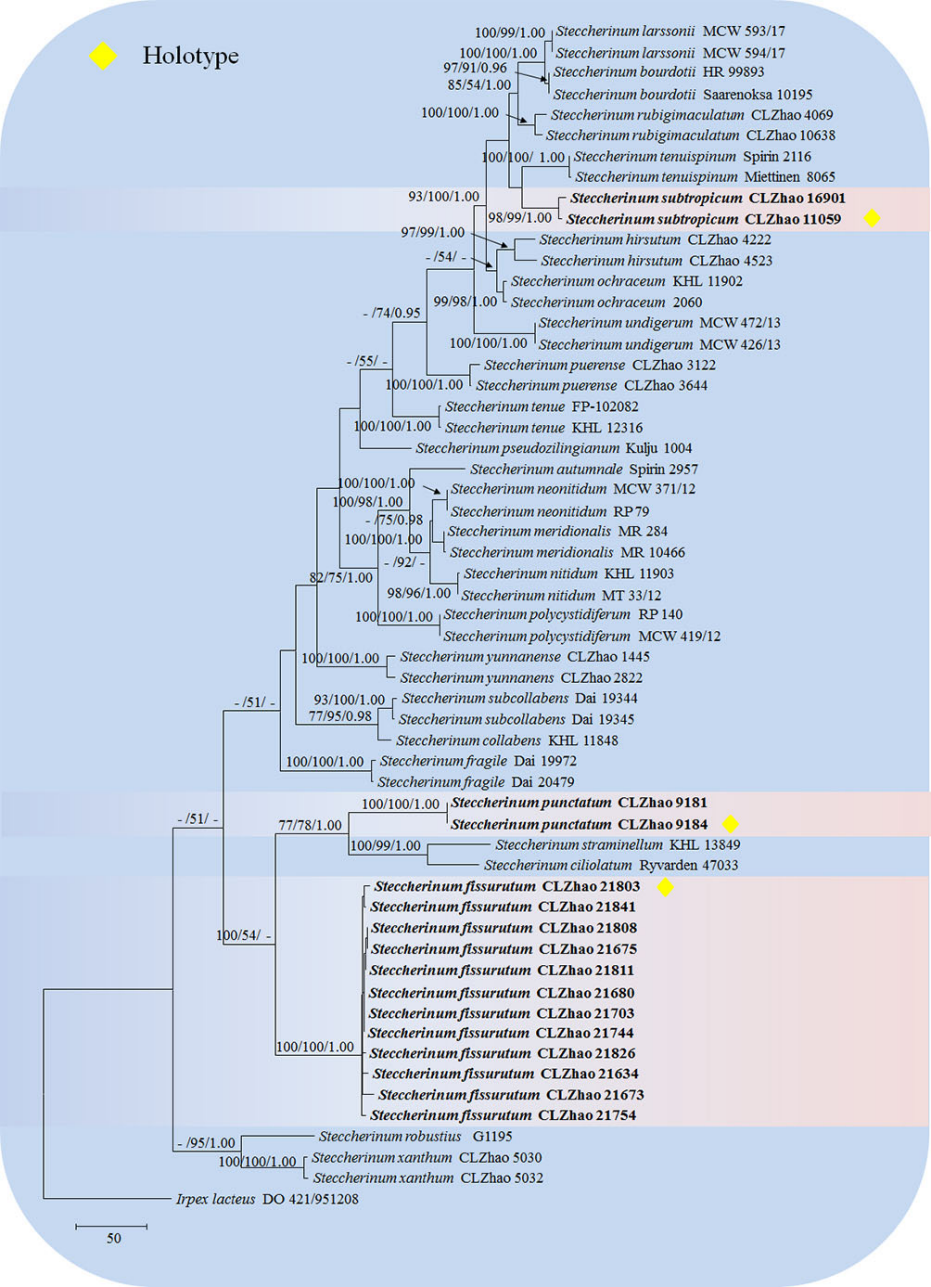
Maximum parsimony strict consensus tree illustrating the phylogeny of three new species and related species in *Steccherinum* based on ITS+nLSU sequences. Branches are labeled with maximum likelihood bootstrap values higher than 70%, parsimony bootstrap values higher than 50% and Bayesian posterior probabilities more than 0.95 respectively.

The sequences were aligned in MAFFT version 7 (Katoh et al., 2019) using the G-INS-i strategy. The alignment was adjusted manually using AliView version 1.27 (Larsson, 2014). The dataset was aligned first and then ITS and nLSU sequences were combined with Mesquite version 3.51. Alignment datasets were deposited in TreeBASE (submission ID 29889). Sequence of *Climacocystis borealis* (Fr.) Kotl. & Pouzar obtained from GenBank was used as an outgroup to root trees in the ITS+nLSU analysis in the family Steccherinaceae (**Figure 1**), and *Irpex lacteus* (Fr.) Fr. was used as an outgroup in the ITS+nLSU analysis in the genus *Steccherinum* (**Figure 2**) (Dong et al., 2022).

Maximum parsimony (MP), maximum likelihood (ML) and Bayesian inference (BI) analyses were applied to the combined three datasets following previous study (Zhao and Wu, 2017), and the tree construction procedure was performed in PAUP* version 4.0b10 (Swofford, 2002). All characters were equally weighted and gaps were treated as missing data. Trees were inferred using the heuristic search option with TBR branch swapping and 1000 random sequence additions. Max-trees were set to 5000, branches of zero length were collapsed, and all parsimonious trees were saved. Clade robustness was assessed using bootstrap (BT) analysis with 1000 replicates (Felsenstein, 1985). Descriptive tree statistics-tree length (TL), consistency index (CI), retention index (RI), rescaled consistency index (RC), and homoplasy index (HI) were calculated for each maximum parsimonious tree generated. The multiple sequence alignment was also analyzed using maximum likelihood (ML) in RAxML-HPC2 through the Cipres Science Gateway (Miller et al., 2012). Branch support (BS) for ML analysis was determined by 1000 bootstrap replicates.

MrModeltest 2.3 (Nylander, 2004) was used to determine the best-fit evolution model for each data set for Bayesian inference (BI), which was performed using MrBayes 3.2.7a with a GTR+I+G model of DNA substitution and a gamma distribution rate variation across sites (Ronquist et al., 2012). A total of 4 Markov chains were run for 2 runs from random starting trees for 2.8 million generations for ITS+nLSU in Steccherinaceae (**Figure 1**), and 1.7 million generations for ITS+nLSU in *Steccherinum* (**Figure 2**) with trees and parameters sampled every 1000 generations. The first one-fourth of all generations was discarded as burn-in. The majority rule consensus tree of all remaining trees was calculated. Branches were considered as significantly supported if they received maximum likelihood bootstrap value (BS) >70%, maximum parsimony bootstrap value (BT) >70%, or Bayesian posterior probabilities (BPP) >0.95.

## Results

### Molecular phylogeny

The ITS+nLSU dataset (**Figure 1**) included sequences from 82 fungal specimens representing 50 species. The dataset had an aligned length of 2257 characters, of which 1304 characters are constant, 237 are variable and parsimony uninformative, and 716 are parsimony informative. Maximum parsimony analysis yielded 36 equally parsimonious trees (TL = 3992, CI = 0.3885, HI = 0.6115, RI = 0.6621, and RC = 0.2572). The best model for the ITS+nLSU dataset estimated and applied in the Bayesian analysis was GTR+I+G (lset nst = 6, rates = invgamma; prset statefreqpr = dirichlet (1,1,1,1)). Bayesian analysis and ML analysis resulted in a similar topology to MP analysis with an average standard deviation of split frequencies = 0.007830 (BI), and the effective sample size (ESS) across the two runs is the double of the average ESS (avg ESS) = 182.

The phylogram inferred from the ITS+nLSU rDNA gene regions (**Figure 1**) showed that sixteen genera nested into the family Steccherinaceae as *Antella* Miettinen, *Antrodiella* Ryvarden & I.johans, *Atraporiella* Ryvarden, *Butyrea* Miettinen, *Elaphroporia* Z.Q. Wu & C.L. Zhao, *Etheirodon* Banker, *Flaviporus* Murrill, *Frantisekia* Spirin & Zmitr, *Junghuhnia* Corda, *Loweomyces* (Kotl. & Pouzar) Jülich, *Metuloidea* G. Cunn, *Mycorrhaphium* Maas Geest, *Nigroporus* Murrill, *Steccherinum*, *Trullella* Zmitr and *Xanthoporus* Audet, in which three new species *Steccherinum fissurutum*, *S. punctatum* and *S. subtropicum* grouped into genus *Steccherinum*.

The ITS+nLSU dataset (**Figure 2**) included sequences from 57 fungal specimens representing 27 species. The dataset had an aligned length of 2068 characters, of which 1465 characters are constant, 168 are variable and parsimony-uninformative, and 435 are parsimony-informative. Maximum parsimony analysis yielded 5000 equally parsimonious trees (TL = 1640, CI = 0.5213, HI = 0.4787, RI = 0.7996, RC = 0.4169). Best model for the ITS+nLSU dataset estimated and applied in the Bayesian analysis was GTR+I+G (lset nst = 6, rates = invgamma; prset statefreqpr = dirichlet (1,1,1,1). Bayesian analysis and ML analysis resulted in a similar topology to MP analysis with an average standard deviation of split frequencies = 0.009192 (BI), and the effective sample size (ESS) across the two runs is the double of the average ESS (avg ESS) = 198.

The phylogenetic tree (**Figure 2**) inferred from ITS+nLSU sequences covered 26 species of *Steccherinum*, which demonstrated that *S. subtropicum* was sister to *S*. *enuispinum*; *S*. *fissurutum* formed a monophyletic lineage; *S. punctatum* grouped with a clade comprised *S. straminellum* (Bres.) Melo and *S. ciliolatum* (Berk. & M.A. Curtis) Gilb. & Budington.

### Taxonomy

**Figure 3:**
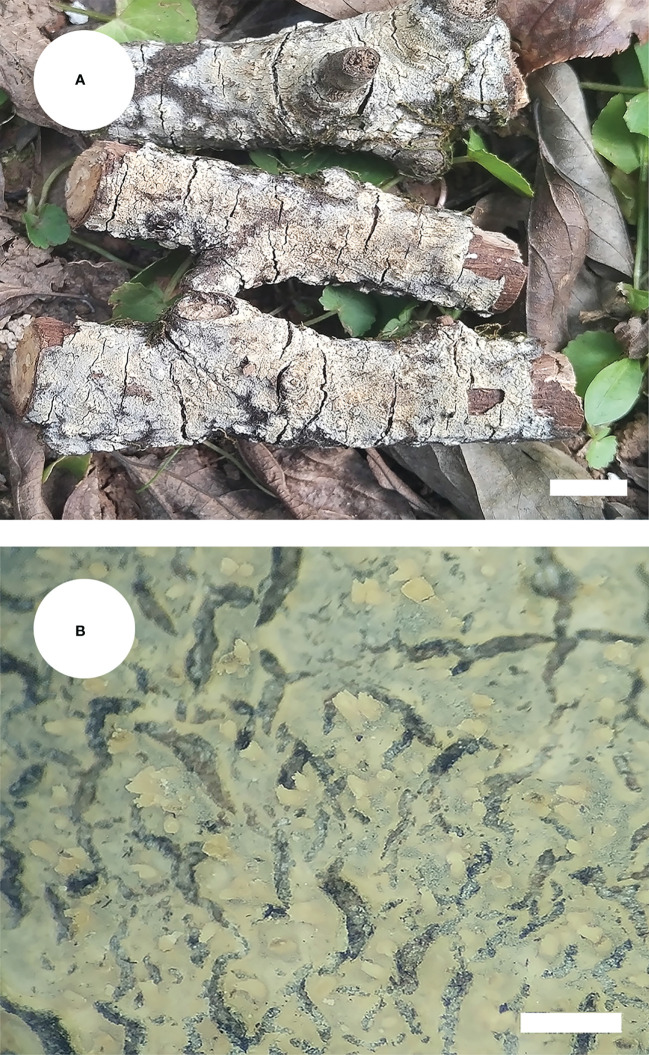
Basidiomata of *Steccherinum fissurutum* (holotype). Bars: **(A)** 1 cm; **(B)** 0.5 mm.

**Figure 4:**
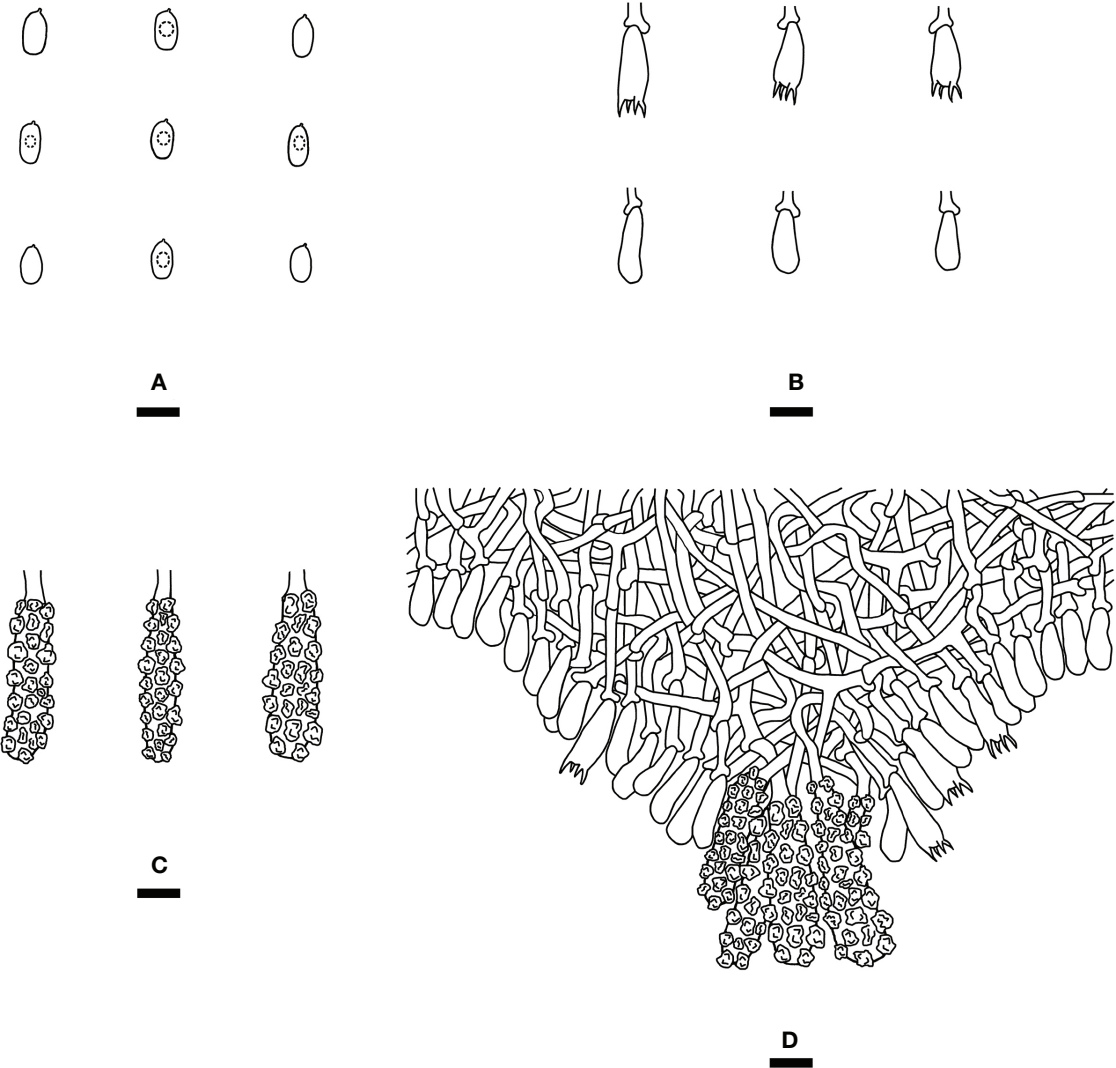
Microscopic structures of *Steccherinum fissurutum* (drawn from the holotype). **(A)** Basidiospores. **(B)** Basidia and basidioles. **(C)** Skeletocystidia. **(D)** A section of hymenium. Bars: **(A)** 5 µm; **(B–D)** 10 µm.

*Steccherinum fissurutum* J.H. Dong & C.L. Zhao, sp. nov. **Figures 3**, **4**.

Hierarchical information: Fungi, Dikarya, Basidiomycota, Agaricomycotina, Agaricomycetes, Polyporales, Steccherinaceae, *Steccherinum.*

*MycoBank no.*: MB 846499.

Diagnosis: differs from other *Steccherinum* species by its white to buff, cracked, subceraceous, grandinoid hymenial surface, a monomitic hyphal system with clamped generative hyphae and cylindrical basidiospores measuring 4.5–6.0 × 2.5–3.0 µm.

Holotype—China. Yunnan Province, Lijiang, Heilongtan Park, Xiangshan, GPS coordinates 26°53′ N, 100°13′ E, altitude 2, 400 m asl., on the fallen branch of angiosperm, leg. C.L. Zhao, 21 July 2021, CLZhao 21803 (SWFC).

Etymology—*fissurutum* (Lat.): referring to the cracked hymenophore surface of the type specimens.

*Basidiomata*: Annual, resupinate, adnate, cracked, subceraceous, without odor or taste when fresh, becoming brittle upon drying, up to 10 cm long, up to 2 cm wide, 50–150 µm thick. Hymenial surface grandinoid, aculei 3–5 per mm, the length of aculei up to 0.2 mm, white (60) when fresh, turning to white (60) to buff (13) upon drying. Sterile margin white, 0.5 mm wide.

*Hyphal system*: Monomitic, generative hyphae with clamp connections, colorless, thin-walled, frequently branched, interwoven, 2.5–3.5 µm in diam. IKI–, CB–, tissues unchanged in KOH.

*Hymenium*: Skeletocystidia numerous in the aculei, strongly encrusted in the obtuse apex, 26.5–36 × 6.5–9.5 µm; cystidioles absent. Basidia clavate, with 4 sterigmata and a basal clamp connection, 12.5–16.5 × 4.5–7 µm; basidioles dominant, in shape similar to basidia, but slightly smaller.

*Basidiospores:* Cylindrical, colorless, thin-walled, with one oil drop inside, IKI–, CB–, 4.5–6.0 × 2.5–3.0 µm, L = 5.23 µm, W = 2.79 µm, Q = 1.75–1.98 (n = 180/6).

*Type of rot*: White rot.

*Additional specimens examined (paratypes)*: CHINA, Yunnan Province, Lijiang, Heilongtan Park, Xiangshan, GPS coordinates 26°53′ N, 100°13′ E, altitude 2, 400 m asl., on the fallen branch of angiosperm, leg. C.L. Zhao, 21 July 2021, CLZhao 21634, 21673, 21675, 21680, 21703, 21744, 21754, 21808, 21811, 21826, 21841 (SWFC).

**Figure 5:**
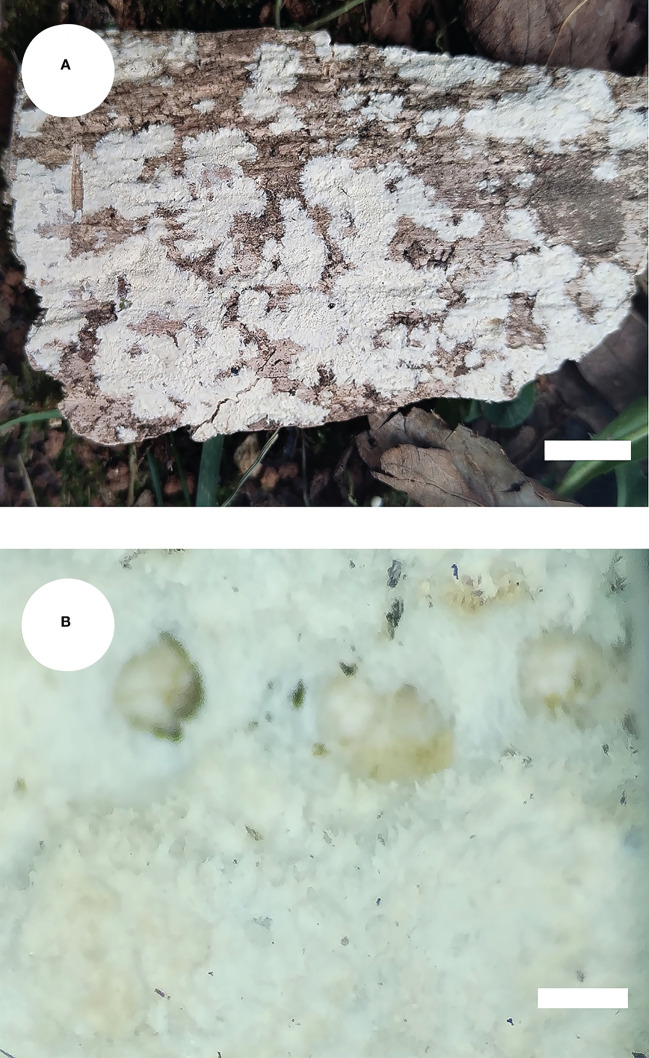
Basidiomata of *Steccherinum punctatum* (holotype). Bars: **(A)** 1 cm; **(B)** 0.5 mm.

**Figure 6:**
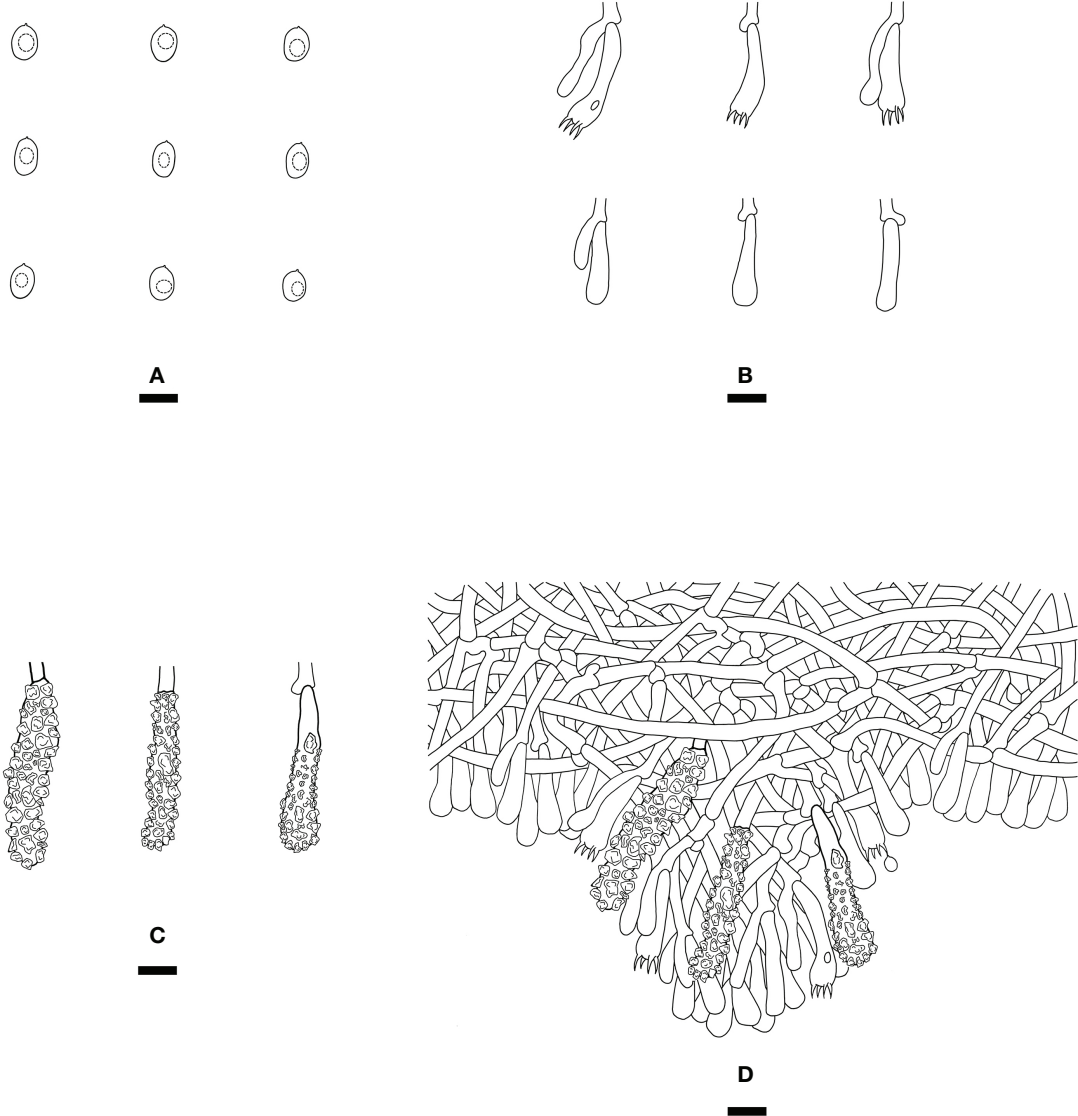
Microscopic structures of *Steccherinum punctatum* (drawn from the holotype). **(A)** Basidiospores. **(B)** Basidia and basidioles. **(C)** Skeletocystidia. **(D)** A section of hymenium. Bars: **(A)** 5 µm; **(B–D)** 10 µm.

*Steccherinum punctatum* J.H. Dong & C.L. Zhao, sp. nov. **Figures 5**, **6**.

Hierarchical information: Fungi, Dikarya, Basidiomycota, Agaricomycotina, Agaricomycetes, Polyporales, Steccherinaceae, *Steccherinum.*

*MycoBank no.*: MB 846500.

Diagnosis: differs from other *Steccherinum* species by its cream to buff, punctate, grandinoid hymenial surface, a monomitic hyphal system with clamped generative hyphae and ellipsoid basidiospores measuring 3.6–4.5 × 2.6–3.4 µm.

Holotype—China. Yunnan Province, Yuxi, Xinping County, Jinshan Primeval Forest Park, GPS coordinates 24°07′ N, 101°99′ E, altitude 2, 300 m asl., on the stump of angiosperm, leg. C.L. Zhao, 2 January 2019, CLZhao 9184 (SWFC).

Etymology—*punctatum* (Lat.): referring to the punctate hymenophore surface.

*Basidiomata*: Annual, resupinate, adnate, punctate, soft leathery, without odor or taste when fresh, becoming leathery upon drying, up to 15 cm long, up to 5 cm wide, 50–100 µm thick. Hymenial surface grandinoid, aculei 5–9 per mm, the length of aculei up to 0.1 mm, white (60) when fresh, turning to cream (21) to buff (13) upon drying. Sterile margin cream, 0.5 mm wide.

*Hyphal system*: Monomitic, generative hyphae with clamp connections, colorless, thin-walled, frequently branched, interwoven, 3–4.5 µm in diam. IKI–, CB–, tissues unchanged in KOH.

*Hymenium*: Skeletocystidia numerous, thin-walled, cylindrical, strongly encrusted in the surface and almost entirely, 36–47 × 7.5–12 µm; cystidioles absent. Basidia subclavate to barrel, with 4 sterigmata and a basal clamp connection, 23–27 × 5.5–7.5 µm; basidioles dominant, in shape similar to basidia, but slightly smaller.

*Basidiospores:* Ellipsoid, colorless, thin-walled, smooth, with one oil drop inside, IKI–, CB–, 3.6–4.5(–4.7) × 2.6–3.4 µm, L = 4.00 µm, W = 2.88 µm, Q = 1.37–1.42 (n = 60/2).

*Type of rot*: White rot.

*Additional specimen examined (paratype)*: CHINA, Yunnan Province, Yuxi, Xinping County, Jinshan Primeval Forest Park, GPS coordinates 24°07′ N, 101°99′ E, altitude 2, 300 m asl., on the stump of angiosperm, leg. C.L. Zhao, 2 January 2019, CLZhao 9181 (SWFC).

**Figure 7:**
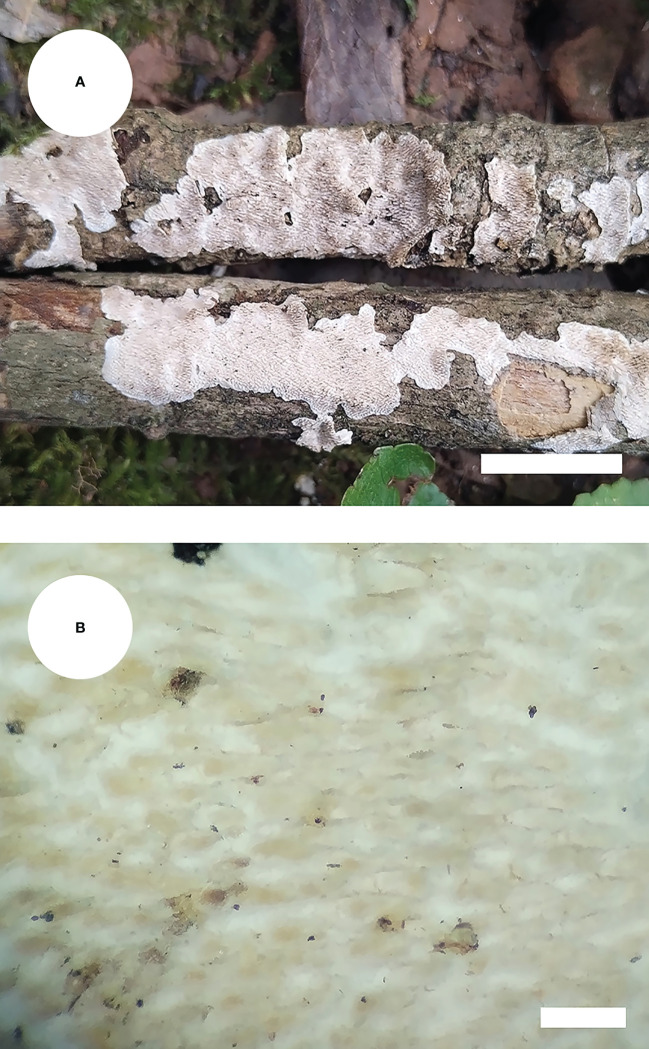
Basidiomata of *Steccherinum subtropicum* (holotype). Bars: **(A)** 1 cm; **(B)** 0.5 mm.

**Figure 8:**
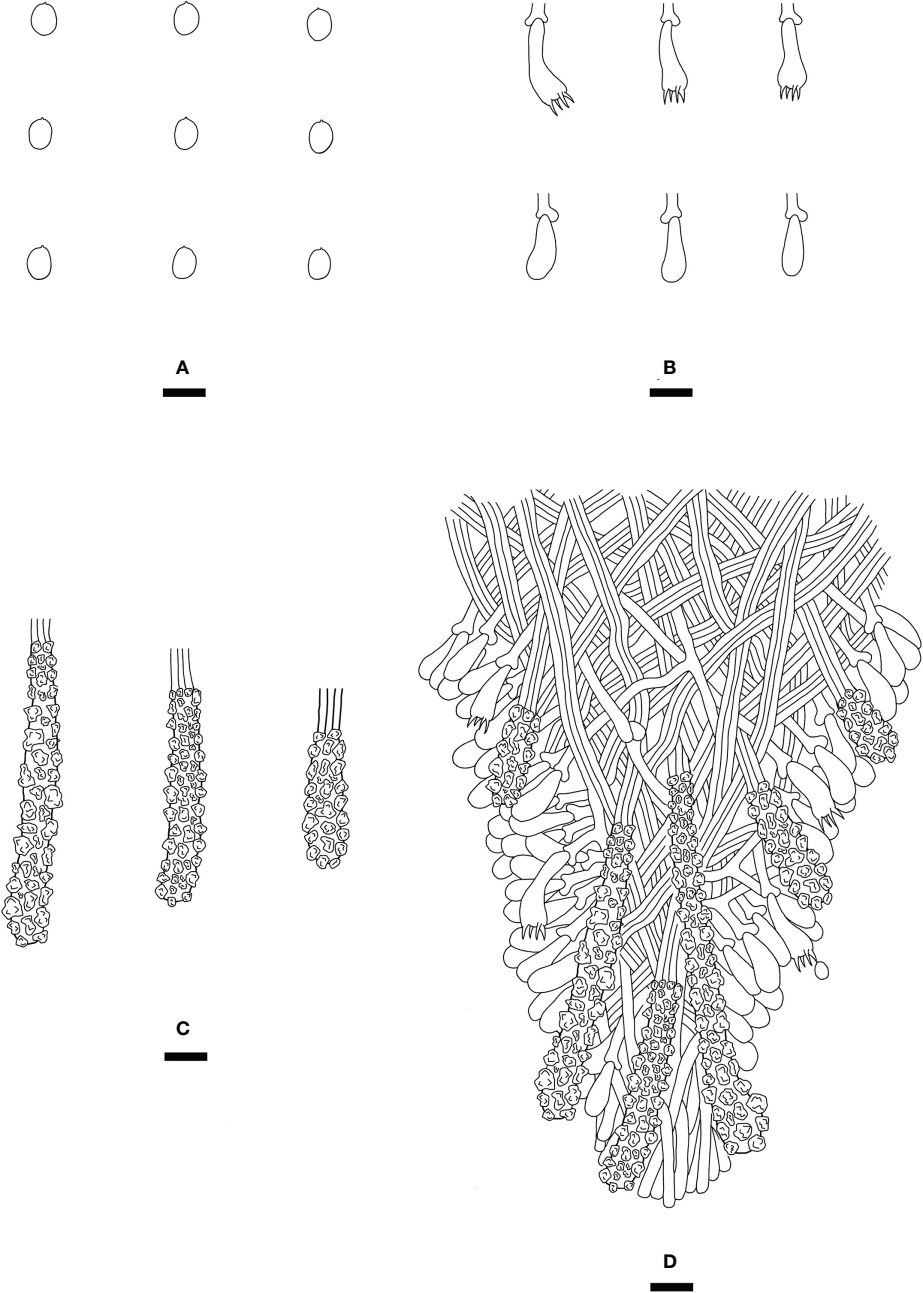
Microscopic structures of *Steccherinum subtropicum* (drawn from the holotype). **(A)** Basidiospores. **(B)** Skeletocystidia. **(C)** Basidia and basidioles. **(D)** A section of hymenium. Bars: **(A)** 5 µm; **(–D)** 10 µm.

*Steccherinum subtropicum* J.H. Dong & C.L. Zhao, sp. nov. **Figures 7**, **8**.

Hierarchical information: Fungi, Dikarya, Basidiomycota, Agaricomycotina, Agaricomycetes, Polyporales, Steccherinaceae, *Steccherinum.*

*MycoBank no.*: MB 846501.

Diagnosis: differs from other *Steccherinum* species by its pink to lilac, effuse-reflexed, odontioid hymenial surface, a dimitic hyphal system with clamped generative hyphae and ellipsoid basidiospores measuring 2.8–3.4 × 2.0–2.7 µm.

Holotype—China. Yunnan Province, Wenshan, Xichou County, Xiaoqiaogou National Nature Reserve, GPS coordinates 23°22′ N, 104°47′ E, altitude 1700 m asl., on the fallen branch of angiosperm, leg. C.L. Zhao, 15 January 2019, CLZhao 11059 (SWFC).

Etymology—*subtropicum* (Lat.): referring to distribution (subtropical zone) of the type specimens.

*Basidiomata*: Annual, effuse-reflexed, without odor or taste when fresh, becoming leathery upon drying, up to 6 cm long, up to 1.5 cm wide, 100–150 µm thick. Hymenial surface odontioid, aculei 5–7 per mm, the length of aculei 0.5–1 mm long, fresh pink (27) when fresh, turning to rose (28) to lilac (48) upon drying. Sterile margin cream, 0.5–1 mm wide.

*Hyphal system*: Dimitic, generative hyphae with clamp connections, colorless, thin-walled, branched, more or less interwoven, 2.3–3.5 µm in diam. Skeletal hyphae colorless, thick-walled, 3.5–4.5 µm diam; all hyphae IKI–, CB–, tissues unchanged in KOH.

*Hymenium*: Skeletocystidia numerous strongly encrusted in the obtuse apex, 20–82 × 5.5–10 µm; cystidioles absent. Basidia clavate, with 4 sterigmata and a basal clamp connection, 14.5–20 × 4–6 µm; basidioles dominant, in shape similar to basidia, but slightly smaller.

*Basidiospores:* Ellipsoid, colorless, thin-walled, IKI–, CB–, 2.8–3.4 × 2.0–2.7 µm, L = 3.00 µm, W = 2.31 µm, Q = 1.24–1.37 (n = 60/2).

*Type of rot*: White rot.

*Additional specimen examined (paratype)*: CHINA, Yunnan Province, Wenshan, Xiaojie Town, Laojunshan National Nature Reserve, GPS coordinates 22°56′ N, 104°37′ E, altitude 2500 m asl., on the fallen branch of angiosperm, leg. C.L. Zhao, 15 January 2019, CLZhao 16901 (SWFC).

## Discussion

In the present study, three new species, *Steccherinum fissurutum*, *S. punctatum* and *S. subtropicum* are described based on phylogenetic analyses and morphological characters.

Phylogenetically, seven clades were found in Polyporales: the residual polyporoid clade, the phlebioid clade, the antrodia clade, the tyromyces clade, the fragiliporia clade, the core polyporoid clade and the gelatoporia clade (Binder et al., 2005; Binder et al., 2013). Miettinen et al. (2012) employed the molecular systematics of *Steccherinum* and related genera *Antrodiella*, and *Junghuhnia* utilizing sequences of the gene regions ITS, nLSU, mtSSU, ATPase subunit 6 (*atp6*), RNA polymerase II second largest subunit (*rpb2*), and translation elongation factor 1-alpha (*tef1*), to reveal that at least 16 transitions have taken place between poroid and hydnoid hymenophore types within the family Steccherinaceae. In the present study, based on the sequences of the gene regions ITS and nLSU (**Figure 1**), three new species, *S. fissurutum*, *S. punctatum* and *S. subtropicum* nested within the genus *Steccherinum*. Amplifying ITS and nLSU genes across genus *Steccherinum* (**Figure 2**), *S*. *fissurutum* formed a monophyletic lineage; *S. punctatum* grouped with a clade comprised *S. straminellum* and *S. ciliolatum*; *S. subtropicum* was sister to *S*. *tenuispinum* Spirin, Zmitr. & Malysheva. However, morphologically, *S. straminellum* differs from *S. punctatum* by having the dimitic hyphal system and narrower basidiospores (3.5–4.5 × 2.0–2.2 µm; Melo, 1995); *S. ciliolatum* is distinguished from *S. punctatum* by having narrowly ellipsoid to cylindrical basidiospores (4–4.5 × 2.2–2.5 µm; Maas Geesteranus, 1974). *S. tenuispinum* differs from *S. subtropicum* by its fimbriate rhizomorphs and longer aculei (1–4 mm; Spirin et al., 2007).

Morphologically, *Steccherinum fissurutum* resembles *S. litschaueri* and *S. ciliolatum* in having cylindrical basidiospores. However, *S. litschaueri* is distinguished from *S. fissurutum* by its rhizomorphic margin and narrower basidiospores (4.5–5.5 × 2.0–2.2 µm; Bernicchia and Gorjón, 2010). *Steccherinum ciliolatum* differs in having longer aculei (up to 1.5 mm) and longer basidia (18–22 × 4.5–6 µm; Maas Geesteranus, 1974).

*Steccherinum punctatum* is similar to *S. hydneum* Rick ex Maas Geest., *S. tenuispinum* and *S. yunnanense* in having leathery hymenophore. However, *S. hydneum* differs from *S. punctatum* by its longer aculei (2–3 mm) and wider basidiospores (4.2–5.0 × 3.6–4.1 µm; Yuan and Dai, 2005b); *S. tenuispinum* differs from *S. punctatum* in having whitish to dirty-ochraceous hymenial surface and narrower basidia (12–24 × 3.5–4.8 µm; Spirin et al., 2007); *S. yunnanense* differs in its fimbriate margin and shorter basidia (10.5–15 × 5–6 µm; Dong et al., 2022). *Steccherinum punctatum* resembles *S. aggregatum* Hjortstam & Spooner, *S. fragile* and *S. xanthum* in having a monomitic hyphal system. However, *S. aggregatum* is distinguished from *S. punctatum* by having longer cystidia (100–150 × 10–12 µm) and smaller basidia (15–20 × 4–5 µm; Hjortstam et al., 1990); *S. fragile* differs in having the fragile basidiomata and smaller basidiospores (2.8–3.1 × 2.1–2.2 μm; Liu and Dai, 2021). *Steccherinum xanthum* is distinguished from *S. punctatum* in having smaller basidia (10–19.3 × 3–5.2 μm; Wu et al., 2021b).

*Steccherinum subtropicum* is similar to *S. hydneum*, *S. oreophilum* Lindsey & Gilb. and *S. rubigimaculatum* in the effuse-reflexed basidiomata. However, *S. hydneum* differs from *S. subtropicum* by its cinnamon buff hymenial surface and larger basidiospores (4.2–5.0 × 3.6–4.1 µm; Yuan and Dai, 2005b). *Steccherinum oreophilum* differs in its cottony hymenophore and larger basidiospores (5–6.5 × 3–3.2 µm; Bernicchia and Gorjón, 2010); *S. rubigimaculatum* differs in having rust hymenial surface and longer basidiospores (3.5–5 × 2.5–3.5 µm; Wu et al., 2021a); *S. subtropicum* resembles *S. fragile*, *S. ochraceum* and *S. robustius* (J. Erikss. & S. Lundell) J. Erikss. in having ellipsoid basidiospores. However, *S. fragile* is distinguished from *S. subtropicum* in having a monomitic hyphal system and shorter basidia (13–14 × 4.0–4.5 µm; Liu and Dai, 2021). *S. ochraceum* differs in its ocherous hymenial surface and longer cystidia (100 × 7–10 µm; Bernicchia and Gorjón, 2010). The species *S. robustius* is distinguished from *S. subtropicum* by its fimbriate margin and longer basidiospores (3.5–5 × 2.5–3 µm; Bernicchia and Gorjón, 2010).

Fungi are one of the most diverse groups of organisms on Earth and play a crucial role in ecosystem processes and functions (Hyde, 2022). New DNA sequencing techniques have revolutionized the researches of fungal taxonomy and diversity, in which about 150 thousand species of fungi have been described (Hyde, 2022). Wood decaying fungi have been studied intensively in recent years (Bernicchia and Gorjón, 2010; Dai, 2011; Cui et al., 2019; Guan et al., 2020; Wang and Zhao, 2021; Westphalen et al., 2021; Wu et al., 2021a; Wu et al., 2021b; Wu et al., 2021b; Luo and Zhao, 2022; Luo et al., 2022; Qu et al., 2022; Wu et al., 2022a; Wu et al., 2022b), but the hydnoid species in the order Polyporales are still not well investigated in China, especially in the subtropics and tropics. In the present study, three new species, *Steccherinum fissurutum*, *S. punctatum* and *S. subtropicum* spp. nov. were found in subtropics, which enriches the fungal diversity of East Asia.

### Key to species of *Steccherinum* sensu lato from China

1. Hyphal system monomitic in subiculum······························2

1. Hyphal system dimitic in subiculum····································8

2. Basidiospores <2 μm wide··········*Mycorrhaphium adustum*

2. Basidiospores >2 μm wide······················································3

3. Skeletocystidia absent····························*Steccherinum fragile*

3. Skeletocystidia present·····························································4

4. Aculei >1mm long·············································*S. aggregatum*

4. Aculei <1 mm long···································································5

5. Aculei <0.3 mm long, basidiospores with oil drops··········6

5. Aculei >0.3 mm long, basidiospores without oil drops···································································*Cabalodontia queletii*

6. Basidia >20 μm long··········································*S. punctatum*

6. Basidia <20 μm long································································7

7. Cystidia>35 μm long, basidiospores ellipsoid····*S. xanthum*

7. Cystidia<35 μm long, basidiospores Cylindrical·······································································**·** *S. fissurutum*

8. Skeletocystidia absent···········································*S. hirsutum*

8. Skeletocystidia present····························································9

9. Skeletocystidia subulate, apex acute···································10

9. Skeletocystidia clavate, apex blunt······································12

10. Basidiospores >5 μm wide, aculei >1.5 mm long···················································································*S. oreophilum*

10. Basidiospores <5 μm wide, aculei <1.5 mm long·········11

11. Basidiomata surface reddish to brick, basidiospores <2 μm wide················································································*S. laeticolor*

11. Basidiomata surface white to buff, basidiospores >2 μm wide····················································································*S. subulatum*

12. Basidiomata resupinate························································13

12. Basidiomata effused-reflexed··············································16

13. Basidiomata with broom-like rhizomorphs···················································*Etheirodon fimbriatum*

13. Basidiomata without broom-like rhizomorphs···············14

14. Basidiospores <2 μm wide·································*S. mukhinii*

14. Basidiospores >2 μm wide··················································15

15. Aculei <0.5 mm long, aculei <4 per mm··················································································*S. tenuissimum*

15. Aculei >0.5 mm long, aculei >4 per mm·····*S. ochraceum*

16. Sterile margin fimbriate······················································17

16. Sterile margin not fimbriate··············································18

17. Basidiospores <3.5 μm wide···························*S. yunnanense*

17. Basidiospores >3.5 μm wide····························*S. elongatum*

18. Basidiospores <4 μm long·················································19

18. Basidiospores >4 μm long·················································25

19. Aculei <2 mm long·····························································20

19. Aculei >2 mm long·····························································23

20. Aculei >0.5 mm long··························································21

20. Aculei <0.5 mm long··························································22

21 Basidiospores <2 μm wide····························*S. subcollabens*

21 Basidiospores >2 μm wide····························*S. subtropicum*

22. Basidiospores subcylindrical to allantoid················································································*S. puerense*

22. Basidiospores ellipsoid······································*S. cremicolor*

23. Aculei 3–4 mm long, pileus margin sharp ····································································*Metuloidea murashkinskyi*

23. Aculei up to 2 mm long, pileus margin blunt···············24

24. Basidiospores >1.5 μm wide····························*S. rawakense*

24. Basidiospores <1.5 μm wide························*S. confragosum*

25. Basidiospores subglobose····················································26

25. Basidiospores ellipsoid························································27

26. Aculei <2 mm long, basidiospores with a normal guttule or not··············································································*S. subglobosum*

26. Aculei >2 mm long, basidiospores with a distinct guttule···················································································*S. hydneum*

27. Basidia <11 μm long····························*S. rubigimaculatum*

27. Basidia >11 μm long····························································28

28. Basidiospores >3 μm wide································*S. bourdotii*

28. Basidiospores <3 μm wide···················································29

29. Aculei >0.5 mm long, pinkish buff to clay buff························································································*S. robustius*

29. Aculei <0.5 mm long, cream to pale buff·······················································································*S. ciliolatum*

## Data availability statement

The datasets presented in this study can be found in online repositories. The names of the repository/repositories and accession number(s) can be found in the article/supplementary material.

## Author contributions

Conceptualization, C-LZ; methodology, C-LZ and J-HD; software, C-LZ and J-HD; validation, C-LZ and J-HD; formal analysis, C-LZ and J-HD; investigation, C-LZ, Z-LZ, and J-HD; resources C-LZ; writing—original draft preparation, C-LZ, J-HD, X-CZ, and J-JC; writing—review and editing, C-LZ and J-HD; visualization, C-LZ and J-HD; supervision, C-LZ; project administration, C-LZ; funding acquisition, C-LZ and Z-LZ. All authors have read and agreed to the published version of the manuscript.
